# Sex- and age-dependent effects of chronic corticosterone exposure on depressive-like, anxiety-like, and fear-related behavior: Role of amygdala glutamate receptors in the rat

**DOI:** 10.3389/fnbeh.2022.950000

**Published:** 2022-09-23

**Authors:** Megan L. Bertholomey, Vidhya Nagarajan, Dana M. Smith, Mary M. Torregrossa

**Affiliations:** ^1^Department of Psychology and Neuroscience Program, Allegheny College, Meadville, PA, United States; ^2^Department of Psychiatry, University of Pittsburgh, Pittsburgh, PA, United States; ^3^Department of Neurobiology, University of Pittsburgh, Pittsburgh, PA, United States

**Keywords:** female, stress, adolescence, adulthood, anxiety, fear

## Abstract

Persistent glucocorticoid elevation consistent with chronic stress exposure can lead to psychopathology, including mood and anxiety disorders. Women and stress-exposed adolescents are more likely to be diagnosed with mood disorders, suggesting that sex and age are important factors in determining vulnerability, though much remains to be determined regarding the mechanisms underlying this risk. Thus, the aim of the present experiments was to use the chronic corticosterone (CORT) exposure paradigm, a model of depression-like behavior that has previously been established primarily in adult males, to determine the mood-related effects of CORT in female and adolescent rats. Depression- and anxiety-like effects in adulthood were determined using the sucrose preference (SPT), the forced swim test (FST), the elevated plus maze, and fear conditioning. Basolateral amygdala (BLA) and medial prefrontal cortex (mPFC) glutamate receptor subunit levels were then measured. In a subsequent experiment, adult male and female rats were tested for the effects of pharmacological activation (via AMPA) or inhibition (via NBQX) of AMPA receptors in the BLA on behavior in the FST. Overall, females showed reduced anxiety- and depressive-like behaviors relative to males. However, females treated with CORT in adolescence, but not adulthood, had increased immobility in the FST, indicative of depression-like behavior. In contrast, CORT did not alter behavior in adolescent-treated males, though the previously reported depression-like effect of adult CORT exposure was observed. Control females had higher expression of the AMPA receptor subunits GluA1 and GluA2/3 selectively in the BLA relative to males. Adolescent CORT treatment, however, decreased BLA GluA1 and GluA2/3 expression in females, but increased expression in males, consistent with the direction of depression-like behavioral effects. Male and female rats also demonstrated opposing patterns of response to BLA AMPA receptor modulation in the FST, with AMPA infusion magnifying the sex difference of decreased immobility in females. Overall, these experiments show that increased glutamate receptor function in the BLA may decrease the risk of developing depressive-like behavior, further supporting efforts to target glutamatergic receptors for the treatment of stress-related psychiatric disorders. These findings also support further focus on sex as a biological variable in neuropsychiatric research.

## Introduction

Persistently elevated glucocorticoid levels consistent with chronic stress have detrimental effects on the brain, resulting in an increased risk for psychopathology in vulnerable populations (McEwen, 2004; Joëls, 2011; Juster et al., 2011; Merkulov et al., 2017). Dysregulation of brain stress systems is implicated in the etiology of anxiety and mood disorders (de Kloet et al., 2005; McEwen, 2005), which are twice as prevalent in women as men (Kessler, 2003; McLean et al., 2011). Adversity early in life is also associated with a higher incidence of mood and anxiety disorders as a consequence of high circulating glucocorticoid levels during a critical period of neuronal development (Andersen and Teicher, 2008; Lupien et al., 2009; Eiland and Romeo, 2013). Taken together, this highlights that both females and adolescents represent populations vulnerable to the effects of chronic stress on neurobiological functioning. Though there are a growing number of studies aimed at elucidating both sex and age as factors in the expression of anxiety- and depression-related symptoms, much remains to be determined regarding the mechanisms underlying these complex relationships.

To model anxiety and depression in animals, chronic stress procedures are commonly used (Willner, 2005; Gourley and Taylor, 2009). While age-dependent effects of stress more consistently show altered emotional functioning in adolescent-exposed animals relative to adults (McCormick, 2010; Eiland and Romeo, 2013), sex differences in these responses are more variable. For example, exposure to chronic mild stress (CMS) normalized the enhanced depressive-like behavior in adult females compared to males in the forced swim test (FST), but reduced sucrose preference (a measure of anhedonia) to a greater degree in males than females (Dalla et al., 2005). Other studies utilizing CMS (Bielajew et al., 2003) and chronic injection of the major stress hormone in rodents, corticosterone (Kalynchuk et al., 2004) also indicate that adult female rats are less sensitive to the effects of chronically elevated glucocorticoids in producing depressive-like behavior than males. Studies comparing across sex when stress is administered in adolescence are also variable. Chronic restraint, immunological stressor exposure, or severe sporadic stress in adolescence produced anxiogenic effects in male rats tested in adulthood, while these manipulations (Ariza Traslavina et al., 2014) or exposure to CMS had either no effect or were anxiolytic in female rats (Pohl et al., 2007; Ariza Traslavina et al., 2014). However, in the Pohl et al. (2007) study, both stress types produced an anhedonic effect only in females tested in adulthood. In contrast, social isolation stress in adolescence produced acute increases in depressive-like behavior in female rats, but these effects were not evident in adulthood (Mathews et al., 2008). Similarly, chronic variable stress in adolescence increased depressive-like behavior in females when tested in adolescence (Dearing et al., 2021) and adulthood (Caruso et al., 2017), but both sexes showed increased in anxiety-like behavior when measured in early adulthood (Caruso et al., 2017). Finally, exposure to chronic mixed modality stressors (social isolation, social defeat, and restraint) in adolescence produced anxiogenic and depressive-like responses in female rats both acutely and in adulthood, while having no effect in males (Bourke and Neigh, 2011). These findings show that the type of stressor used and time of testing have significant effects on behavioral responses to stress as a function of sex, and have led to a very confusing and inconsistent literature.

To minimize the variability induced by different modalities of stressors, the chronic corticosterone (CORT) model is a non-invasive method for producing persistently elevated glucocorticoid levels consistent with mild stressor exposure (Gourley and Taylor, 2009). This model elicits depressive-like effects in the sucrose preference test and FST (Gourley and Taylor, 2009) and maladaptive processing of fear-related memory (Gourley et al., 2009; Monsey et al., 2014), but has no effects on anxiety-like behavior (e.g., the elevated plus maze), when tested in adult males. Chronic CORT exposure in adolescence produces increases in impulsive choice in male rats (Torregrossa et al., 2012), enhances cue-related alcohol craving in female rats (Bertholomey et al., 2016) and enhances alcohol-, but not sucrose-motivated behavior in male rats (Bertholomey et al., 2018) when tested in adulthood, indicating that elevated CORT levels during this critical developmental period can lead to long-lasting changes in pathological behavior. Importantly, the chronic CORT model has been used to determine the persistent effects of a brief, fixed duration of elevated glucocorticoid levels, as testing occurs long after the CORT has washed out (>10 days), and circulating CORT levels have returned to baseline. Thus, measures are taken while the animals are not under the influence of exogenous glucocorticoids. However, only recently have sex differences in the effects of chronic CORT been studied, and none, to our knowledge, have explored both sex- and age-dependent effects of chronic CORT on anxiety- and depressive-like behavior. In studies conducted in mice, chronic CORT exposure had mixed results: one study showed that females, but not males, showed greater immobility in the FST following CORT exposure (Berger et al., 2019), whereas another showed increases in anxiety- and depressive-like behavior in males, but not females, using a similar CORT procedure (Caradonna et al., 2022). However, it should be noted that both of these studies were conducted in adult animals.

Therefore, the goals of the present experiments were to determine whether 3 weeks of chronic CORT exposure in either adolescence or adulthood in male and female rats would differentially affect anxiety-like, depressive-like, and fear-related behavior after a prolonged washout period in both age groups, corresponding to adulthood in adolescent-exposed rats. Further, as glutamatergic systems are implicated in the pathophysiology of anxiety and depression (Riaza Bermudo-Soriano et al., 2012; Duman, 2014), chronic CORT exposure has been shown to alter the expression of NMDA (GluN2B) and AMPA (GluA1, GluA2-3) receptor subunits in the ventromedial prefrontal cortex (vmPFC) and GluA1 in the lateral amygdala (Gourley et al., 2009; Monsey et al., 2014), and notably, sex-specific increases in *Gria1* are evident in the PFC of chronically stressed female mice (Barko et al., 2019) and human women with major depressive disorder (Gray et al., 2015), expression of these glutamate receptor proteins was determined.

## Materials and methods

### Subjects

Young adult (aged 67–68 postnatal days [p67–68] upon arrival; *n* = 24 females, 24 males for Experiment 1; *n* = 36 females, 32 males for Experiment 2) or adolescent (aged 27–28 postnatal days [p27–28] upon arrival; *n* = 51 females, 44 males; for Experiment 1) Sprague-Dawley Rats (Harlan, Frederick, MD, United States) were housed in pairs and maintained on a 12:12 h light:dark cycle in a temperature- and humidity-controlled animal room. Rats were allowed to acclimate to the colony room for at least 2 days prior to experimental manipulations. Behavioral testing was conducted in adulthood in all experiments (with the exception of the first two sucrose preference tests in adolescent-exposed rats) during the light cycle. Rats were given *ad libitum* access to water and food throughout the experiment except where noted below. All procedures were conducted in accordance with the policies set forth by the University of Pittsburgh Institutional Animal Care and Use Committee and the National Institutes of Health Guidelines on the Care and Use of Laboratory Animals.

### Experiment 1: Effects of adolescent vs. adult chronic corticosterone exposure on depressive-like, anxiety-like, and fear-related behavior in male and female rats

**FIGURE 1:**
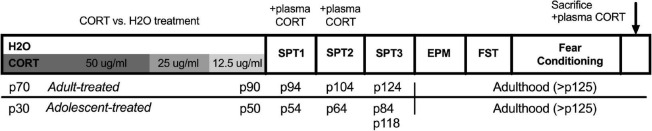
Timeline of experimental procedures for Experiment 1. Adult-treated rats exposed to either CORT or H_2_O as their sole source of fluid in adulthood (p70–90), while adolescent-treated rats were exposed in adolescence (p30–50). All behavioral testing was conducted in adulthood, with the exception of the first two sucrose preference tests in adolescent-exposed rats that took place in the transition from late adolescence to early adulthood. CORT, corticosterone; H_2_O, water; p, postnatal day; SPT, sucrose preference test; EPM, elevated plus maze; FST, forced swim test.

A timeline of the experimental procedures for Experiment 1 can be found in Figure 1. Note: elevated plus maze, forced swim testing, and fear conditioning were conducted in that order within a narrow time frame following sucrose preference testing across all cohorts, but were not conducted on consecutive days. The order of behavioral testing was chosen to expose rats from the least to the most stressful tests to minimize carryover effects. In addition, the timing of the sucrose preference testing (discussed in detail below) was designed to capture anhedonic-like effects as a function of both the timing relative to the CORT exposure as well as age/development.

#### Chronic corticosterone exposure

Separate groups of rats received access to a single bottle of either CORT (4-pregnen-11b,21-diol-3,20-dione21-hemisuccinate, Steraloids, Newport, RI, United States, dissolved in tap water) or water as their sole fluid in their home cage. Fluid intake was measured daily, and body weights were recorded every other day. CORT was administered in decreasing concentrations as follows: 14 days at 50 μg/ml; 3 days at 25 μg/ml; and 3 days at 12.5 μg/ml. The CORT exposure procedure was the same as that previously described and that has been reported to produce circulating CORT levels of greater than 800 ng/ml (Gourley et al., 2008; Gourley and Taylor, 2009). Following CORT exposure, rats were given a 10-day washout period before further testing to allow for the normalization of HPA axis function (Gourley and Taylor, 2009). Rats were exposed to CORT either in adulthood on postnatal days 70–90 (p70–90), or during adolescence on postnatal days 30–50 (p30–50); (Spear, 2000).

#### Corticosterone assays

Tail blood samples were taken immediately following the first two sucrose preference tests, which corresponded to proximal (4 days) and post-washout (14 days) time points following the cessation of CORT exposure. At the end of the experiment, rats were euthanized via rapid decapitation, and blood samples were collected in heparinized 1.5 ml centrifuge tubes. Blood samples were collected during the latter half of the light cycle. Plasma from each time point was analyzed for CORT using commercially available EIA kits (Enzo Life Sciences, Farmingdale, NY, United States) according to the manufacturer’s instructions.

#### Sucrose preference testing

To assess potential sex differences in the anhedonic-like effects of chronic CORT exposure, rats were tested for sucrose preference (SPT) at three time points in adulthood and adolescence: immediately following the cessation of CORT exposure (SPT1; p54 or p94 in adolescents and adults, respectively), following the 10-day washout period (SPT2; p64 or p104), and a later time point corresponding to adulthood in the adolescent-treated rats (SPT3; p84 or p124). Rats were habituated to a solution of 1% (w/v) sucrose in their home cages for 24 h prior to SPT1. Before each test, rats were fluid restricted for 19 ± 2 h. During testing, rats were given 1-h access to one bottle containing sucrose and one bottle containing water in a novel cage. Preference ratios were calculated by determining the amount of sucrose consumed relative to total fluid (sucrose + water) consumed. While 1% is a commonly used concentration in sucrose preference tests (Willner et al., 1996), upon discovering high preference ratios, the concentration of sucrose was reduced to 0.5% (w/v) during SPT3 in the adult CORT-treated rats and in an additional cohort of adolescent CORT-treated rats.

#### Elevated plus maze

To measure anxiety-like behavior, rats were then tested in the elevated plus maze (EPM; MedPC; Pellow et al., 1985). The EPM consisted of two open and two closed arms (50 cm long, closed arms 42 cm high) raised 75 cm from the floor. Rats were placed in the center of the EPM under low illumination for 5 min, and number of entries (all four paws) into and time spent in the open and closed arms were measured. For all behavioral measures (EPM, FST, and fear conditioning), behavior was videorecorded and assessed by raters with no knowledge of the rats’ experimental condition.

#### Forced swim testing

To measure depressive-like behavior, rats were subjected to forced swim testing (FST). Rats were placed in a cylindrical Plexiglas tank filled to 30 cm in 25 ± 1°C water for 15 min (Armario, 2021). Time spent climbing (vigorous movement of all four limbs, with the forelimbs breaking the plane of the water) and immobile (lack of limb movement) was scored by analyzing the blinded video recordings. The tank was drained and refilled between each rat, and rats were dried and placed in holding cages on heating pads after testing.

#### Fear conditioning

Finally, rats were tested in a cued fear conditioning paradigm (Gourley et al., 2009; Monsey et al., 2014). Testing was carried out in standard conditioning chambers housed in sound-attenuating cubicles (MedPC, St. Albans, VT, United States), and the houselight and fan were on during all phases. On Day 1, rats underwent fear conditioning during a 5-min session in Context A, the last 30 s of which consisted of the presentation of a 75 dB tone (cue) that co-terminated with a 1-s, 1.5 mA footshock. All subsequent testing was conducted in the absence of shock. On Day 2, rats were tested for memory recall, wherein they were placed in a novel context (Context B; different chamber walls, floors, and an almond odor) and received a single cue presentation to measure freezing as an indicator of how well fear memory was acquired and consolidated the day before. On Day 3, rats were given a 30-min extinction session in Context B, where they received 12 cue presentations to extinguish the shock-tone memory. On Day 4, rats were subjected to an extinction recall test in parameters identical to Day 3 to test for the efficacy of the extinction training. Freezing (the absence of movement except breathing) was measured during the pre-cue period to test for context generalization, and during each cue presentation to measure conditioned fear.

#### Estrous cycle monitoring

To determine whether any sex differences observed in behavior are due to the estrous cycle in females, vaginal cytology was assessed by gently pipetting 150 μl saline into the vaginal canal and then onto a slide, which was coverslipped and visualized at 200× magnification under a light microscope that same day (Goldman et al., 2007). Male rats were handled in a similar manner to control for handling stress.

#### Western blotting

Western blot analyses were performed in a similar manner to previous reports (Torregrossa et al., 2012). Brains were flash-frozen in isopentane on dry ice, and stored at −80°C until dissection. Brains were cut into 2-mm sections using a metal brain mold, and tissue from several brain regions, including the medial prefrontal cortex (mPFC, consisting of both prelimbic and infralimbic regions) and basolateral amygdala (BLA), were obtained using a tissue punch (1.2 mm diameter, Fine Science Tools, Foster City, CA, United States) and stored at −80°C. Tissue punches were homogenized using a sonicator in a buffer containing 1.37 mM NaCl, 20 mM TrisHCl, 1% Igepal, 10% glycerol, and 1:100 protease inhibitor and phosphatase inhibitor cocktails II and III (Sigma-Aldrich, St. Louis, MO, United States). Protein concentrations in each sample were then quantified using the Pierce bicinchoninic acid assay kit (ThermoScientific, Rockford, IL, United States). Samples were prepared by diluting 30 μg of protein 4:1 in 1% SDS and loading buffer containing 20% glycerol, 2% SDS, and bromophenol blue, and were then boiled for 10 min at 90°C. For Western blotting, samples were loaded onto 10–20% Tris-glycine gradient gels (Invitrogen, Carlsbad, CA, United States) and proteins were separated using electrophoresis. Proteins were then transferred to nitrocellulose membranes and incubated in the following primary antibodies: anti-phospho Ser1303 NR2B (Rb, 1:500, Millipore, Billerica, MA, United States), anti-NR2B (Ms, 1:500, Millipore, Billerica, MA, United States), anti-phospho Ser831 GluR1 (Rb, 1:1000; Abcam, San Francisco, CA, United States), anti-GluR1 (Ms, 1:1000; Millipore, Billerica, MA, United States), anti-GluR2/3 (Rb, 1:500; Millipore, Billerica, MA, United States), and anti-GAPDH (Ms, 1:1000; Millipore, Billerica, MA, United States). Blots were incubated in the following secondary antibodies for analysis on the LI-COR Odyssey Imager: IRDye 800CW anti-rabbit and IRDye 680RD anti-mouse (1:5000; LI-COR Biosciences; Lincoln, NE, United States). Protein bands were identified and quantified using Licor-Odyssey software. Each protein sample was normalized to its respective GAPDH expression within each gel, and bands were further normalized to the average of the male H_2_O-treated group to control for between gel variations.

### Experiment 2: Effects of intra-basolateral amygdala pharmacological manipulation of AMPA receptors on depressive-like behavior in male and female rats

#### Surgery

Rats were injected with 90 mg/kg ketamine and 5 mg/kg xylazine, and then 5 ml of lactated Ringers’ solution 5 mg/kg of Rimadyl (NSAID analgesic) once anesthetized. A ∼2 cm midline incision was made in the scalp, the skull surface was cleared, and guide cannulae (28 gauge, PlasticsOne) aimed 1 mm above the basolateral amygdala (from bregma: male rats: AP −3.0 mm; ML ±5.2 mm; DV −7.9 mm; female rats: AP −2.8 mm; ML ±5.0 mm; DV −7.8 mm) were implanted in the skull. Three stainless steel screws and dental cement anchored the guide cannulae, and stylets were placed therein to maintain patency. Rats were monitored and allowed to recover for at least 10 days postoperatively prior to experimental testing.

#### Microinjections and forced swim testing

Following recovery from surgery, rats were tested for the effects of either (RS)-2-amino-3-(5-methyl-3-oxo-1,2 -oxazol-4-yl)propanoic acid (AMPA), 2,3-Dioxo-6-nitro-1,2,3,4-tetrahydrobenzo[*f*]quinoxaline-7-sulfonamide disodium salt (NBQX; AMPA and NBQX both from Tocris Bioscience via Fisher Scientific, Pittsburgh, PA, United States), or saline, on behavior in the FST. All conditions of forced swim testing, video recording, and scoring are identical to those described above for Experiment 1, with the exception that in this experiment rats received a second, 5-min test. On day 1 (baseline), rats were habituated to the microinjection procedure (removing guide cannulae, handling, exposure to pump and timer noise), and then placed into the tank for a 15-min baseline test. On day 2 (test) rats were randomly assigned to receive microinjections of 0.1 nmol AMPA, 30 nmol NBQX (Nishitani et al., 2014), or an equivalent volume of saline at a rate of 0.5 μl/min/side over 2 min, allowing an additional minute after injection for diffusion. Rats were then taken to the adjacent testing room and placed into the swim tank for 5 min.

#### Statistics

Statistical analyses were conducted using IBM SPSS Statistics v21 (IBM Corporation, Armonk, NY, United States) or GraphPad Prism 6 (GraphPad Software, Inc., La Jolla, CA, United States). For Experiment 1, missing values due to random accidents (e.g., fluid spillage) were interpolated from the day preceding and following that data point. Data points determined to be outliers (exceeding 2 standard deviations above or below the group mean, and/or identified as outliers using stem-and-leaf plots) were substituted with the mean value of that group. These instances were few and happened randomly and equally across all treatment groups. Outcome measures were analyzed using two-way (treatment: CORT vs. H_2_O; sex: male vs. female) or mixed factorial (same between-subjects factors as above, with day as the repeated measure) ANOVAs (*p* < 0.05), followed by Bonferroni *post hoc* comparisons where appropriate. Adolescent and adult CORT-treated rats were analyzed separately as these cohorts were not tested simultaneously. For Experiment 2, 2 (sex: male vs. female) × 3 (treatment: AMPA, NBQX, or saline) factorial ANOVAs (*p* < 0.05, with Bonferroni *post hoc* comparisons where appropriate) were performed for time spent immobile and time spent climbing in the FST. Only rats with confirmed histological placement bilaterally in the BLA were included in the analysis.

## Results

### Experiment 1

#### Females exhibit higher consumption of and plasma levels of corticosterone than males regardless of age

**FIGURE 2:**
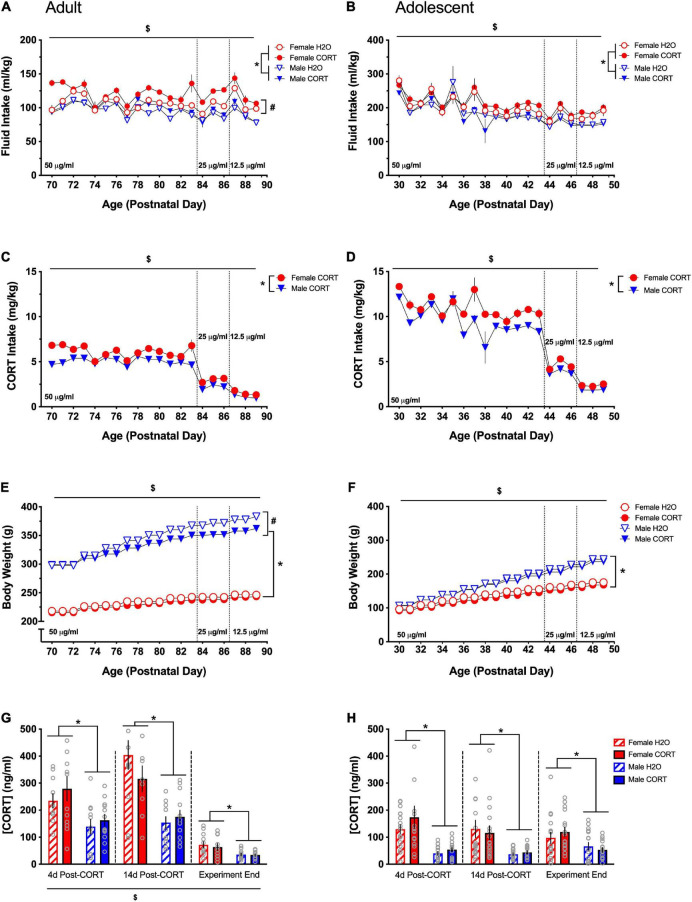
Females exhibit higher consumption of and plasma levels of CORT than males regardless of age. Both adult- **(A)** and adolescent-exposed **(B)** female and CORT-treated rats drank more fluid relative to their body weight (ml/kg) compared to male and H_2_O-treated rats, respectively. Total CORT (mg/kg) intake decreased with titration of the solution concentration, and females received higher doses of CORT than males in both adult **(C)** and adolescent-exposed **(D)** rats. Male rats weighed more than females and gained more weight over time in both adulthood **(E)** and adolescence **(F)**. In adulthood only, CORT treatment reduced body weight in males **(E)**. Adult CORT-treated males weighed significantly, but marginally less than H_2_O-treated controls. Females had higher plasma CORT levels compared to males in adult **(G)** and adolescent **(H)** treated rats at each time point. *p < 0.005 (male vs. female); ^#^*p* < 0.05 (H_2_O vs. CORT); ^$^*p* < 0.05 (main effect of day).

Significant main effects of day, sex, treatment, and day × sex, day × treatment, and treatment × sex interactions were found for fluid intake (ml/kg; Figure 2A [adult] and Figure 2B [adolescent]) during CORT or H_2_O exposure in both age groups [DAY: adults: *F*(19,836) = 30.53, *p* < 0.001, adolescents: *F*(19,475) = 39.48, *p* < 0.001; SEX: adults: *F*(1,44) = 44.19, *p* < 0.001, adolescents: *F*(1,25) = 10.53, *p* = 0.003; TREATMENT: adults: *F*(1,44) = 12.62, *p* = 0.001, adolescents: *F*(1,25) = 6.81, *p* = 0.015; DAY × SEX: adults: *F*(19,836) = 6.17, *p* < 0.001, adolescents: *F*(19,475) = 39.48, *p* < 0.001; DAY × TREATMENT: adults: *F*(19,836) = 2.67, *p* < 0.001; TREATMENT × SEX: adults: *F*(1,44) = 4.92, *p* = 0.032, adolescents: *F*(1,25) = 11.52, *p* = 0.002]. Overall, female rats and CORT-treated rats drank more fluid relative to their body weight compared to male and H_2_O-treated rats, respectively. In adolescent-treated rats, there was a general decrease in ml/kg intake across the treatment period as a function of increased body weight. Follow-up analyses revealed that only in female adult-treated rats did treatment have a significant effect [*F*(1,22) = 15.84, *p* = 0.001], with female CORT-treated rats exhibiting higher and more variable intake across the treatment period relative to H_2_O-treated female rats and male rats. On the other hand, increased intake in females was only evident in adolescent CORT-treated rats [*F*(1,12) = 28.07, *p* < 0.001].

Main effects of day, sex, a day × sex interaction were found for CORT intake in mg/kg (Figure 2C [adult] and Figure 2D [adolescent]) [DAY: adults: *F*(19,418) = 194.47, *p* < 0.001, adolescents: *F*(19,228) = 130.6, *p* < 0.001; SEX: adults: *F*(1,22) = 43.33, *p* < 0.001, adolescents: *F*(1,12) = 24.41, *p* < 0.001; DAY × SEX: adults: *F*(19,418) = 5.41, *p* < 0.001, adolescents: *F*(19,228) = 2.64, *p* < 0.001]. Total CORT intake was reduced as the concentration of CORT in the drinking water was decreased, and females received higher doses of CORT than males. Sex differences in intake in adolescents were most evident during the final week of drinking as the CORT dose was titrated and body weight differences emerged. Overall, females received higher doses of CORT than males.

Main effects of day, sex, and treatment, as well as day × treatment, day × sex, and day × sex × treatment interactions were found for body weight (Figure 2E [adults] and Figure 2F [adolescents]) [DAY: adults: *F*(19,836) = 698.1, *p* < 0.001, adolescents: *F*(19,475) = 2095.6, *p* < 0.001; SEX: adults: *F*(1,44) = 1509.85, *p* < 0.001, adolescents: *F*(1,25) = 141.19, *p* < 0.001; TREATMENT: adults: *F*(1,44) = 9.07, *p* = 0.004; DAY × TREATMENT: adults: *F*(19,836) = 10.22, *p* < 0.001, adolescents: *F*(19,475) = 2.72, *p* < 0.001; DAY × SEX: adults: *F*(19,836) = 145.51, *p* < 0.001, adolescents: *F*(19,475) = 156.43, *p* < 0.001; DAY × SEX × TREATMENT: adults: *F*(19,836) = 7.55, *p* < 0.001]. All rats gained weight across the treatment period, and male rats weighed more than females and gained more weight over time. Overall, CORT-treated rats weighed less than H_2_O-treated rats, though pairwise comparisons indicated that while treatment differences in female rats only approached significance on some treatment days, CORT-treated male rats weighed less than their H_2_O-treated counterparts during the final 14 days of treatment.

Repeated-measures ANOVAs for plasma CORT levels (ng/ml) across the three collection periods (immediately following SPT1, SPT2, and at the end of the experiment) revealed main effects of time [*F*(2,88) = 78.16, *p* < 0.001] in adult-treated rats, while this effect only approached trend-level in adolescent-treated rats (Figure 2G [adults] and Figure 2H [adolescents]). Pairwise comparisons showed that each time point differed from the others, with the greatest overall CORT levels being evident during the second time point and the lowest levels occurring at the end of the experiment. However, a significant main effects of sex was evident on both adult- [*F*(1,44) = 32.15, *p* = 0.001] and adolescent-treated rats [*F*(1,62) = 47.46.71, *p* < 0.001], indicating higher CORT levels in females, and these effects persisted (all *p*s < 0.005), across all time points.

#### Increased sucrose preference in females emerges in adulthood, but is not altered by chronic corticosterone treatment

**FIGURE 3:**
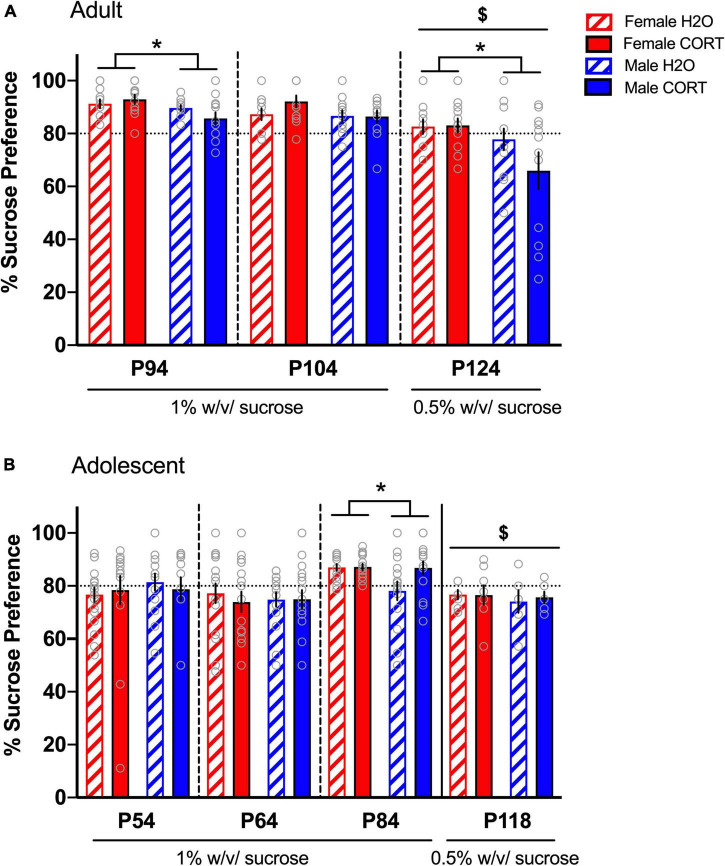
Increased sucrose preference in females emerges in adulthood, but is not altered by chronic CORT treatment. In adult-treated rats **(A)**, female rats showed greater preference than male rats during the first SPT. Sucrose preference was significantly reduced during the final SPT when the sucrose concentration was reduced from 1% to 0.5%, and male CORT-treated rats exhibited a trend (*p* = 0.15) for a reduction in preference compared to their H_2_O-treated counterparts. In adolescent-treated rats **(B)**, sucrose preference was highest on the third test when the rats reached adulthood (p84), and lowest when the concentration was reduced to 0.5%. Only during the third SPT was increased preference in adolescent-treated females evident, indicating that sex differences in hedonic responses emerge in adulthood. *p < 0.05 (male vs. female); ^$^*p* < 0.001 (SPTs at 0.5% vs. SPTs at 1%).

In adult-treated rats, no effects of treatment were found during the first two sucrose preference tests. A main effect of sex was evident during SPT1 [*F*(1,44) = 6.37, *p* = 0.015], with female rats showing a greater preference than male rats (Figure 3A). Since preference ratios exceeded 85% during SPT1-2, potentially leading to a ceiling effect, a lower concentration of sucrose (0.5%) was used for a final SPT (SPT3 in adults). Comparison across the three preference tests [*F*(2,88) = 26.044, *p* < 0.001] and subsequent pairwise comparisons confirmed reduced preference during SPT3 compared to the two prior tests. At this lower sucrose concentration, females still showed greater preference than males [*F*(1,42) = 5.86, *p* = 0.02]. While no significant main effects or interactions with treatment were evident, *a priori* hypotheses based on published data showing reduced sucrose preference in male rats treated with CORT in adulthood (Gourley et al., 2009), we compared across treatment in male rats. Male CORT-treated rats exhibited a trend (*p* = 0.15) for a reduction in preference compared to their H_2_O-treated counterparts, indicating a consistent direction and magnitude of effect as in previous studies, though not statistically significant.

In adolescent-treated rats, no effects of either sex or treatment were evident during SPT1-2. However, a main effect of time [*F*(2,124) = 8.95, *p* < 0.001], revealed that sucrose preference was highest on the third test when the rats reached adulthood (p84), (Figure 3B). Interestingly, this time point also indicated significantly increased preference in adolescent-treated females [*F*(1,58) = 4.14, *p* = 0.046], indicating that sex differences in hedonic responses emerge in adulthood. However, once the concentration of sucrose was reduced (SPT4 in a separate cohort of adolescents), this sex difference disappeared. No effects of CORT exposure were evident in adolescent-treated rats.

#### Anxiety-like behavior is reduced in females relative to males, but is not altered by chronic corticosterone treatment

**FIGURE 4:**
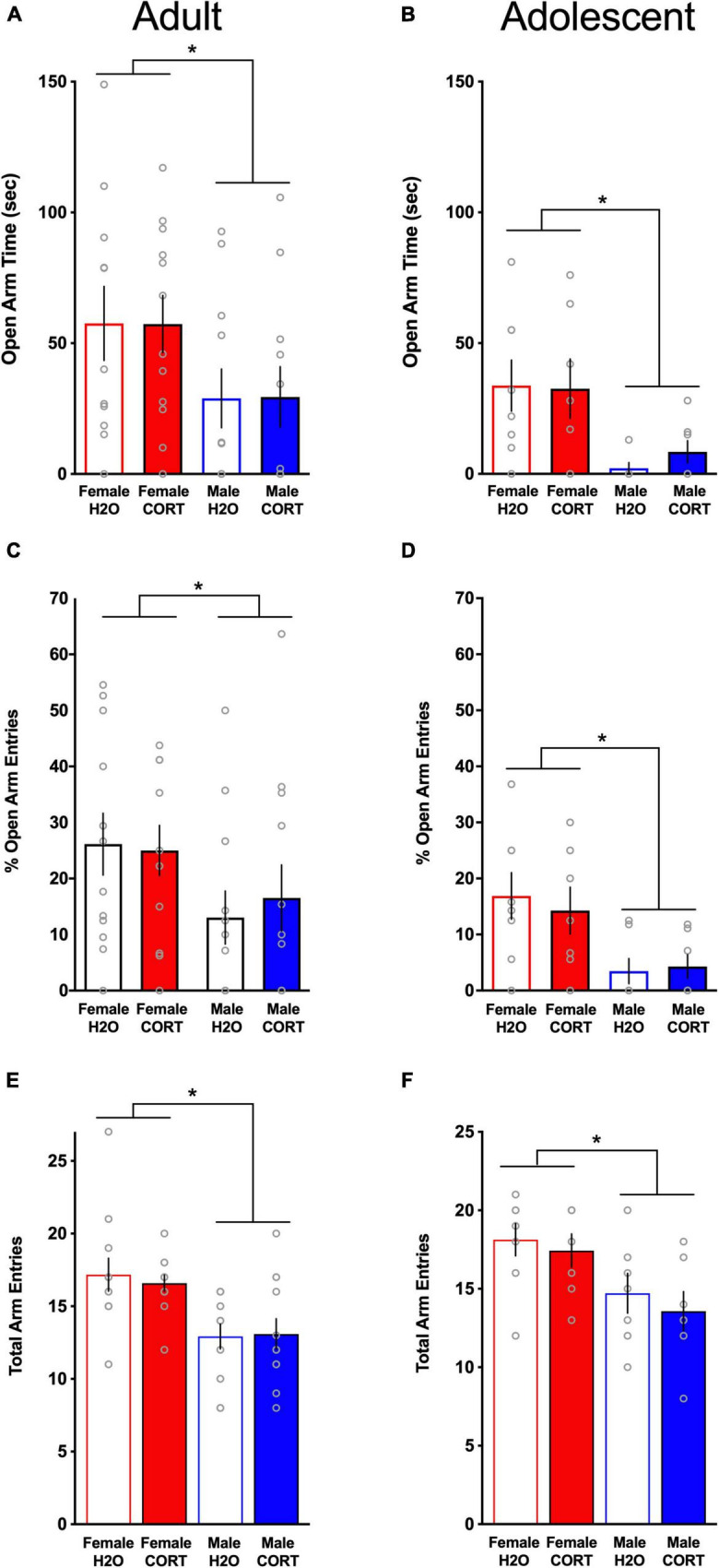
Anxiety-like behavior is reduced in females relative to males, but is not altered by chronic CORT treatment. In both age groups, females spent significantly more time in the open arms [**(A)** adults; **(B)** adolescents], had significantly higher percent open arm entries [**(C)** adults; **(D)** adolescents], and exhibited more total arm entries [**(E)** adults; **(F)** adolescents] compared to males. *p < 0.005 (male vs. female).

In the EPM, female rats demonstrated significantly more time in the open arms (adult: Figure 4A; adolescent: Figure 4B), had greater percent open arm entries (adult: Figure 4C; adolescent; Figure 4D), and made more total arm entries (adult; Figure 4E; adolescent; Figure 4F) compared to male rats, indicating reduced anxiety-like behavior and increased locomotor activity in females [OPEN ARM TIME: adult: *F*(1,39) = 20.75, *p* < 0.001, adolescent: *F*(1,24) = 11.08, *p* = 0.003; OPEN ARM ENTRIES: adult: *F*(1,39) = 11.48, *p* = 0.002, adolescent: *F*(1,25) = 12.35, *p* = 0.002; TOTAL ARM ENTRIES: adult: *F*(1,39) = 15.7, *p* < 0.001, adolescent: *F*(1,25) = 9.95, *p* = 0.004]. Consistent with previous findings in adult-treated males, no effects of CORT were found for anxiety-like behavior in any group. Interestingly, though direct statistical comparisons cannot be made, adolescent CORT-exposed rats tested in adulthood spent less time in the open arms of the EPM relative to adult-exposed/tested rats, possibly indicating age-related increases in stress-related anxiety-like behavior.

#### Corticosterone exposure alters immobility in the FST in a sex- and age-dependent manner

**FIGURE 5:**
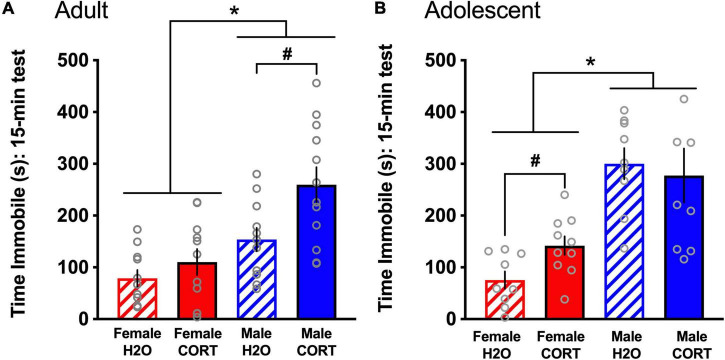
CORT exposure alters depressive-like behavior in a sex- and age-dependent manner. Adult-exposed **(A)** females spent significantly less time immobile than males, and CORT treatment increased immobility in males only. Adolescent-exposed **(B)** females also spent significantly less time immobile than males, but CORT treatment increased immobility in females only. *p < 0.001 (male vs. female); ^#^*p* < 0.01 (H_2_O vs. CORT).

During the FST, both adult- [*F*(1,44) = 20.9, *p* < 0.001; Figure 5A] and adolescent-treated [*F*(1,25) = 29.69, *p* < 0.001; Figure 5B] female rats spent significantly less time immobile than males. In adult-exposed rats, a main effect of treatment [*F*(1,44) = 8.42, *p* = 0.006] and a trend for a sex × treatment interaction (*p* = 0.09) were also found for time spent immobile. Similarly, a treatment effect reached trend level for adolescent-exposed rats (*p* = 0.1). Overall, male and CORT-treated rats showed greater immobility. However, based not only on *a priori* hypotheses of increased depressive-like behavior in adult CORT-treated males and in adolescent CORT-treated females, but also very large effect sizes for the sex differences revealed in the overall ANOVAs (η^2^ = 0.27 [adult-treated]; η^2^ = 0.489 [adolescent-treated]) that may overshadow treatment effects, these trend-level interactions were explored. Independent-samples *t*-tests ran separately for each sex and age revealed that only in male, but not female adult-exposed rats [*t*(22) = −2.84, *p* = 0.01; Figure 5A], and only in female, but not male adolescent-exposed rats [*t*(15) = −4.32, *p* = 0.001; Figure 5B], did CORT treatment significantly increase immobility. No differences as a function of sex, age, or treatment were found for time spent climbing (data not shown).

#### Corticosterone exposure alters fear-related behavior in a sex- and age-dependent manner

**FIGURE 6:**
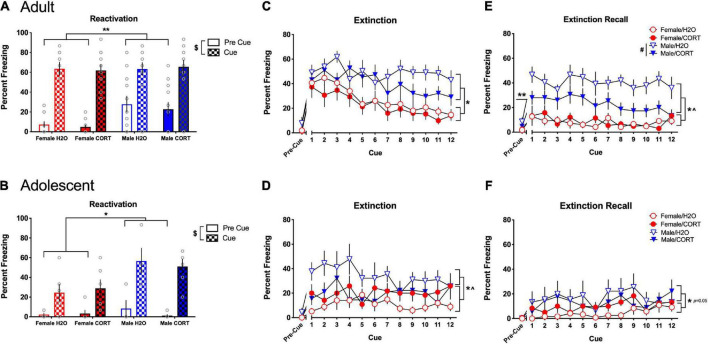
CORT exposure alters fear-related behavior in a sex- and age-dependent manner. In adult-treated rats, all rats increased freezing during the cue compared to the pre-cue period, with males initially showing more freezing than females **(A)**. In adolescent-treated rats, all rats increased freezing during the cue compared to the pre-cue period, with males rats showing more freezing than females when the cue was present **(B)**. Adult-exposed males exhibited significantly higher freezing compared to females during extinction **(C)** and extinction recall **(E)**. Apparent deficits in extinction learning in adult H_2_O-treated males led to significantly higher overall freezing in male rats during the pre-cue period of extinction recall, and H_2_O-treated males showed significantly greater freezing during recall compared to their CORT-treated counterparts. Adolescent-exposed males also exhibited significantly higher freezing compared to females during extinction **(D)**, and a significant sex × treatment interaction indicated that freezing was greater in H_2_O-treated males relative to their CORT-treated counterparts, while female rats showed the opposite pattern. However, no effects of adolescent treatment were evident during recall **(F)**, and only effects of sex reached the level of significance. **p < 0.05 (male vs. female during the pre-cue period); *p < 0.05 (male vs. female during the cue period); ^$^*p* < 0.001 (pre-cue vs. post-cue); ^#^*p* < 0.01 (H_2_O vs. CORT), ^*p* < 0.05 sex × treatment interaction.

During fear reactivation, main effects of cue [*F*(1,40) = 166.59, *p* < 0.001] and sex [*F*(1,40) = 14.05, *p* < 0.001] were found in adult-treated rats, with all rats increasing in freezing during the cue compared to the pre-cue period, and males showing more freezing than females (Figure 6A). Following up on a trend for a cue × sex interaction revealed that significant sex differences were present during the pre-cue period [*F*(1,40) = 13.64, *p* = 0.001], with males showing greater freezing prior to cue exposure. In contrast, while main effects of cue [*F*(1,23) = 63.29, *p* < 0.001] and sex [*F*(1,23) = 14.64, *p* = 0.001] were also found for adolescent-treated rats, with all rats increasing freezing during the cue compared to the pre-cue period, the cue × sex interaction [*F*(1,23) = 8.55, *p* = 0.008] indicated that the sex difference emerged during the cue period, where males showed more freezing than females (Figure 6B).

During extinction, main effects of sex [*F*(1,38) = 20.16, *p* < 0.001] and cue [*F*(11,418) = 7.18, *p* < 0.001] in adult-exposed rats revealed that males exhibited significantly more freezing than females, and all rats showed a general decrease in freezing across the cue presentations (Figure 6C). However, adult males showed attenuated reductions in freezing across extinction, and significantly increased freezing in the pre-cue period of extinction recall [*F*(1,38) = 5.62, *p* = 0.023] (Figure 6E), suggesting a disruption in extinction consolidation in adult males. Further, while significant main effects of sex [*F*(1,38) = 566.57, *p* < 0.001], and treatment [*F*(1,38) = 8.68, *p* = 0.005] during extinction recall showed that male rats and H_2_O-treated rats exhibited more freezing than female and CORT-treated rats, a sex × treatment interaction [*F*(1,38) = 10.42, *p* = 0.003], further revealed that the treatment effect was driven by a significant increase in freezing in H_2_O-treated males relative to their CORT-treated counterparts [*F*(1,18) = 11.38, *p* = 0.003]. Similarly, adolescent-exposed males showed more freezing than females [*F*(1,25) = 6.4, *p* = 0.018] during extinction, and sex × cue [*F*(11,275) = 1.96, *p* = 0.033], and sex × treatment [*F*(1,25) = 6.98, *p* = 0.014] interactions indicated that freezing was greater in H_2_O-treated males relative to their CORT-treated counterparts, while female rats showed the opposite pattern (Figure 6D). Significant, but not systematic effects of cue [*F*(11,264) = 2.38, *p* = 0.008] were found during the recall cue period, and increased freezing in males relative to females reached the level of significance ([*F*(1,24) = 4.27, *p* = 0.05]; Figure 6F).

#### Estrous cycle did not significantly alter anxiety- and depressive-like behavior in females rats

No significant effects of estrous cycle phase was evident for any of the behaviors measured (data not shown). It is possible that these studies, while sufficiently powered to detect behavioral effects as a function of sex and treatment, were underpowered to detect variations as a function of the estrous cycle.

#### Glutamate receptor subunit expression is altered as a function of sex and adolescent corticosterone treatment

**FIGURE 7:**
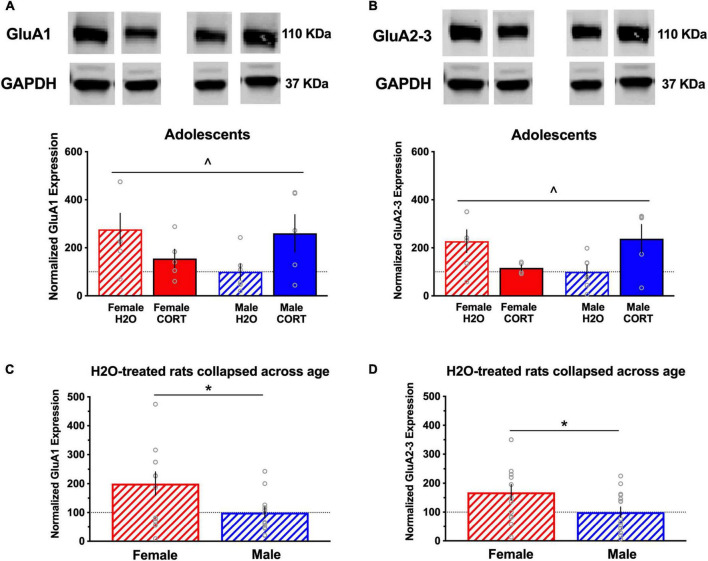
Glutamate receptor subunit expression is altered as a function of sex and adolescent CORT treatment. Representative blots for each treatment group are shown, and a full, uncropped blot can be found in Supplementary Figure S1. Significant sex × treatment interactions were found for expression of the AMPA receptor subunits GluA1 **(A)** and GluA2/3 **(B)** in the BLA of adolescent-exposed rats, with decreased and increased expression in CORT-treated females and males, respectively, relative to their controls. Significant overall sex differences in H_2_O-treated rats were found in BLA levels of GluA1 **(C)** and GluA2-3 **(D)**, indicating significantly increased basal expression in these AMPA receptor subtypes in females compared to males. ^*p* < 0.05 sex × treatment interaction; *p < 0.05 (male vs. female).

In adult-treated rats, no significant differences in the expression of any of the proteins as a function of sex or treatment were found for either region. In contrast, several sex × treatment interactions were found in adolescent treated rats in the BLA (these effects did not reach the level of significance for the mPFC; data not shown). For example, significant sex × treatment interactions were found for GluA1 ([*F*(1,22) = 6.14, *p* = 0.023]; Figure 7A) and GluA2/3 [*F*(1,21) = 8.2, *p* = 0.011]; Figure 7B) expression in the BLA. In addition, overall sex differences in H_2_O-treated rats were found in BLA levels of GluA1 ([*t*(27) = 2.28, *p* = 0.031]; Figure 7C) and GluA2-3 ([*t*(27) = 2.12, *p* = 0.043]; Figure 7D), indicating significantly increased basal expression in these AMPA receptor subtypes in females compared to males.

### Experiment 2

#### AMPA receptors modulate immobility in the forced swim test behavior in a sex-dependent manner

**FIGURE 8:**
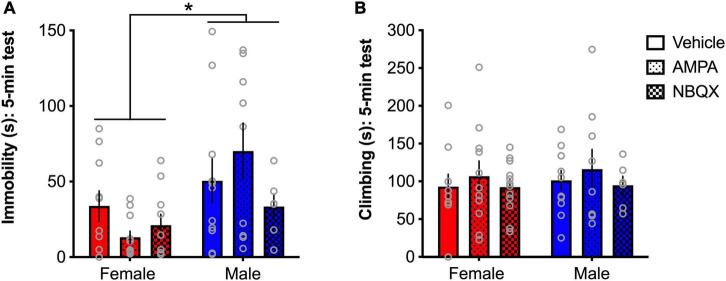
AMPA receptors differentially regulate depressive-like behavior in male and female rats. Females spend significantly less time immobile than males **(A)**, but no differences were found for climbing **(B)**. A trend for a sex × treatment interaction and visual examination of the patterns of effects show that AMPA decreased immobility in females and increased immobility in males. *p < 0.05 (male vs. female).

To further explore the sex differences evident in basal expression of AMPA receptor expression noted in Experiment 1, and other findings of opposing patterns of glutamate-related gene expression in chronically stressed male and female mice (Barko et al., 2019), the effects of intra-BLA AMPA receptor modulation on behavior in the FST in male and female rats were tested in Experiment 2. A main effect of sex was found for time spent immobile in the FST ([*F*(1,51) = 8.66, *p* = 0.049]; Figure 8A), with females spending less time immobile than males, consistent with findings from Experiment 1. While a sex × treatment interaction only approached significance (*p* = 0.1), visual examination of the patterns of effects demonstrate clear sex-dependent outcomes, with AMPA magnifying the sex difference in males and females indicated by reduced immobility in females and increased immobility in males. As such, no main effects of treatment were found for immobility. Further, no main effects of, or interactions involving any of the factors, were found for climbing in the FST (Figure 8B).

## Discussion

The results of the present experiments suggest that sex and age of exposure are significant factors in the effects of prior chronic CORT exposure on mood-related behaviors. Importantly, sex differences in general were more pronounced and pervasive than any effects of CORT treatment. Females consistently exhibited a greater preference for sucrose, reduced anxiety-like, depressive-like and fear-related behavior, and elevated GluA1 and GluA2/3 expression in the BLA compared to males. CORT exposure increased immobility in the FST in adolescent-treated females and decreased BLA GluA1-2/3 expression. CORT exposure produced anhedonic and depressive-like effects in adult males, similar to previously published studies (Gourley and Taylor, 2009), while having no effect in females. Adult CORT exposure did not alter glutamate receptor subtype expression in the BLA. However, increased GluA1-2/3 expression in the BLA of adolescent CORT-treated males may have been protective against the development of depressive-like behavior. Female rats, who show higher basal expression of BLA AMPA (GluA) receptors than males, showed reductions in immobility in the FST following activation of these receptors, whereas male rats tended to show opposing effects, with AMPA activation increasing, and blockade reducing, immobility in the FST.

Our findings are consistent with a number of investigations showing reduced anxiety- and depressive-like behavior in female animals compared to males (Solomon and Herman, 2009; Simpson and Kelly, 2012; Colom-Lapetina et al., 2017); however, the mechanisms underlying these sex-related effects are still unclear. Studies focused on sex differences in stress-related behaviors point to overlapping stress and gonadal hormone systems, though circulating hormone levels do not completely explain difference between males and females. For example, though the present and previous studies show higher plasma CORT levels in females (Kitay, 1961; Weinstock et al., 1998), they generally exhibit less anxiety- and depression-like behavior, suggesting that behavioral differences are not solely driven by absolute levels of circulating stress hormones. In addition, while cumulative CORT intake (mg/kg) was higher in adolescent- versus adult-exposed rats, CORT-associated changes in behavior as a function of age were sex-dependent in the present study. Though hormone fluctuations across the estrous cycle have been shown to modulate anxiety-like behavior (Marcondes et al., 2001) and cued fear conditioning (Milad et al., 2009; Rey et al., 2014; Gruene et al., 2015b), with higher estradiol levels in females being associated with reduced anxiety and enhanced fear memory extinction consolidation relative to lower estradiol levels in females and in males, ovarian hormones have little effect in the FST (Barros and Ferigolo, 1998; Kokras et al., 2017; Caradonna et al., 2022). Importantly, chronic stress procedures have been shown to disrupt cycling in females (Konkle et al., 2003; Dalla et al., 2005), suggesting that future studies should monitor whether chronic CORT exposure leads to similar disruptions in circulating gonadal hormones. However, a recent study showed that various types of chronic stress exposure did not significantly affect gonadal hormones (Anderson et al., 2020). In addition, though estrous was monitored on test days in the current study, the sample size was not sufficient to determine differences in behavior across estrous cycle phase; however, the variability in the behavioral outcome measures in females was not demonstrably larger than in males, consistent with Becker et al. (2016), suggesting that fluctuating gonadal hormone levels in females are not the primary drivers of the sex differences evident in these studies. Rather, our findings point to alterations in glutamatergic systems (discussed below) that provide a promising mechanism for understanding the resilience to stress-related behaviors in females. Nonetheless, several lines of evidence show that males and females exhibit different behavioral responses and strategies, and suggests that measuring outcomes validated in male-only studies may not provide a complete picture of sex differences in these behaviors (Shansky and Murphy, 2021). For example, female rats tend to show more darting behaviors in response to fear-conditioned cues, rather than the male-typical freezing (Gruene et al., 2015a), and males are more likely to exhibit head shakes in the FST compared to females (Kokras et al., 2017). While these particular behaviors were not measured in the present study, future experiments aimed at determining stress-related sex differences in depressive-like and fear-related behavior should consider broadening their observations. In addition, despite being a common test of antidepressant drug effects, it is acknowledged that the validity of the FST in measuring “depressive-like behavior” has been challenged, due in part to differing interpretations of the observed behavior as either active or passive coping, which may differ as a function of sex (Bogdanova et al., 2013; Kokras et al., 2015).

Sex differences in the effects of chronic CORT treatment were also modulated by age of exposure, with adolescent, but not adult CORT-treated females and adult, but not adolescent CORT-treated males significantly increasing immobility in the FST and tending to increase freezing during the extinction of conditioned fear relative to controls. These results are consistent with the predictions of increased sensitivity to chronic stress exposure in depressive-like behavior in adolescent females and adult males based on prior studies (Gourley and Taylor, 2009; Bourke and Neigh, 2011). However, the finding that adult CORT-treated males were less impaired in the extinction of conditioned fear relative to H_2_O-treated controls contrasts with a previous study (Monsey et al., 2014). The opposing results could be due to differences in the proximity of testing relative to the CORT exposure period (2 vs. 4+ weeks post-CORT), and/or the multiple behavioral tests that were conducted prior to fear conditioning in the present study. Future investigation into the precise mechanism by which CORT exposure leads to dysregulation of glucocorticoid and other systems leading to differential behavioral responses as a function of age and sex may uncover targets that can be manipulated to prevent chronic stress-related pathological behavior.

Importantly, our findings demonstrate a potential role for glutamate systems in altering depressive-like behavior as a function of sex and age of CORT exposure. The mPFC and BLA share reciprocal glutamatergic projections, and glutamate functioning in this stress-sensitive circuit has been implicated in the pathophysiology of anxiety- and fear-related (Riaza Bermudo-Soriano et al., 2012) and depressive-like (Duman, 2014) behavior. Accordingly, prior studies have shown that glutamate receptor subtype expression is significantly altered by adult CORT exposure in males (Gourley et al., 2009; Monsey et al., 2014). In the present study, alterations in glutamate receptor subtype expression mapped onto changes in depressive-like behavior. Females showed significantly higher expression of GluA1 and GluA2/3 in the BLA than males, which corresponded with reduced behavioral despair. This female “resilience” was reversed by adolescent CORT treatment, evidenced by decreased pGluN2B and GluA1-2/3 protein expression in the BLA and increased immobility in the FST. In contrast, adolescent CORT-treated males showed increased expression of these proteins and were resistant to changes in FST behavior. Thus, there appears to be an inverse relationship between BLA GluA1-2/3 expression and depressive-like behavior, paralleling previous studies showing pro-resilience and antidepressant-associated up-regulation of GluA2 in the nucleus accumbens after chronic social stress (Vialou et al., 2010).

Interestingly, other studies have shown increased expression of glutamate-related genes, like *Gria1*, in female mice exposed to unpredictable chronic mild stress, relative to males (Barko et al., 2019), indicating that differences across species, or across gene expression and protein concentrations, may not be parallel. However, given findings of opposite molecular signatures of major depressive disorder have been found in men and women (Seney et al., 2018), and baseline differences in AMPA glutamate receptor (GluA) expression in the BLA of our male and female rats in Experiment 1, this inverse relationship was explicitly explored in Experiment 2. Notably, pharmacological activation of AMPA receptors tended to produce an antidepressant-like effect in females, whereas inhibition of AMPA receptors using NBQX tended to produce an antidepressant-like effect in males, while not affecting behavior in females. One possibility for this interesting effect could be that due to increased basal expression of GluA receptors in the BLA of females, a higher dose of NBQX is needed to sufficiently alter behavior. Decreased sensitivity to systemically administered NBQX in females has been demonstrated in alcohol drinking studies showing that the highest dose of NBQX (30 mg/kg) reduced binge-like alcohol drinking in male mice, while having no effect in female mice (Bauer et al., 2020). Another possibility is that since NBQX blocks both AMPA and kainate receptors, kainate-mediated effects may differ as a function of sex. To our knowledge, sex differences in kainate receptor expression or pharmacology has not been measured in adult animals. As such, a deeper exploration of sex differences in dose- and receptor-specific modulation of glutamate receptors would be important targets for future research.

There is evidence for involvement of GluRs in depressive-like behavior, and importantly, sex differences in these effects have been found. While the antidepressant-like effects of the NMDA receptor antagonist, ketamine, have been shown to involve actions at GluN2B (Miller et al., 2014), importantly, AMPA receptors (Maeng et al., 2008), and GluA1 in particular (Zanos et al., 2016), are crucial to the antidepressant efficacy of ketamine. Thus, increased sensitivity to ketamine in females (Franceschelli et al., 2015; Zanos et al., 2016) could be due to higher GluA1 expression in the BLA, and increased sensitivity to the antidepressant effects of AMPA receptor activation. However, the degree to which gonadal hormones influence glutamatergic receptor expression, and may explain the sex differences evident in the present study, is unclear. For example, while one study showed that the basal and lateral parts of the amygdala show greater inhibition during low estradiol phases (diestrus) and high estradiol phases (proestrus), respectively (Blume et al., 2017), another indicated that GluR1 and GluR2/3 expression is not changed by gonadal hormone manipulations in either sex, though other sexually dimorphic brain regions like the hypothalamus did show upregulation of these receptors following hormone replacement with either testosterone in males, or estradiol in females (Diano et al., 1997). Interestingly, the enhanced effects of ketamine in females appear to be mediated by ovarian hormones (Dossat et al., 2018; Saland et al., 2022), and estrogen plus ketamine or its metabolites ((2R,6R)-HNK and (2S,6S)-HNK) had additive effects on increasing *Gria1-4* expression, suggesting that estradiol interacts with AMPA receptors to enhance the effects of ketamine (Ho et al., 2018). This finding may shed light on a potential mechanism of the therapeutic potential of ketamine in treating depression in females, though further studies in neurons is warranted.

The present results confirm that chronic CORT exposure in adult males produces depression-like effects, and extends these findings to adolescent CORT-treated females. Increased expression of AMPA receptor subunits in the BLA are associated with resilience to maladaptive behavior in females and adolescent CORT treated males. Therefore, sex and age of chronic stress exposure are critical determinants to subsequent behavioral outcomes that model psychopathology. Further, our findings point to glutamate signaling in the BLA as a critical mediator of these responses and provide additional evidence that drugs that target AMPA receptors may be particularly useful in reducing depressive symptoms in at-risk populations, including females.

## Data availability statement

The raw data supporting the conclusions of this article will be made available by the authors, without undue reservation.

## Ethics statement

This animal study was reviewed and approved by University of Pittsburgh Institutional Care and Use Committee.

## Author contributions

MB and MT designed the study, analyzed the data, and wrote the manuscript. MB, VN, and DS carried out the study. MB created the figures. All authors provided ongoing feedback on the experimental procedures and contributed to manuscript revision, read, and approved the submitted version.

## Supplementary material

Supplementary Figure S1 Uncropped blots from which representative bands in Figure 7 are derived. Samples with boxes around the labels in the gel map and arrows pointing to the bands correspond to the cropped images in Figure 7 for adolescent BLA expression of GluA1 **(a)**, GluA2/3 **(b)**, and GAPDH **(c)**.
