# Supplementary figures and images for: Sex- and age-dependent effects of chronic corticosterone exposure on depressive-like, anxiety-like, and fear-related behavior: Role of amygdala glutamate receptors in the rat
Source: Front Behav Neurosci. 2022 Sep 23;16:950000. doi: 10.3389/fnbeh.2022.950000 (PMC9537815; doi:10.3389/fnbeh.2022.950000)

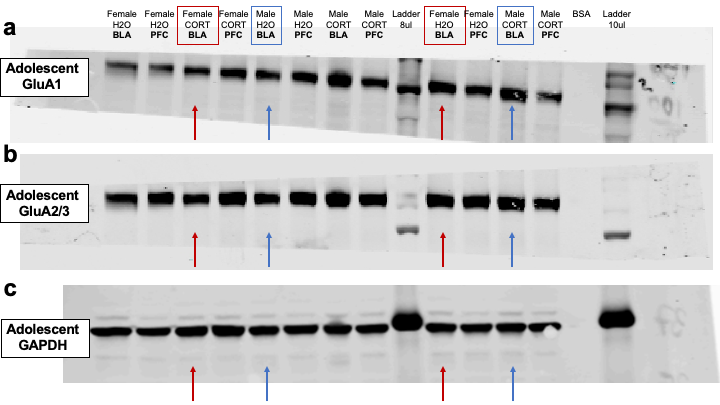

Supplement: Supplementary Figure S1 — Uncropped blots from which representative bands in Figure 7 are derived. Samples with boxes around the labels in the gel map and arrows pointing to the bands correspond to the cropped images in Figure 7 for adolescent BLA expression of GluA1 (a), GluA2/3 (b), and GAPDH (c). [file Image_1.TIFF]
